# Dr. William Garner Sutherland (1873-1954): A Pioneer in Cranial Osteopathy

**DOI:** 10.7759/cureus.95241

**Published:** 2025-10-23

**Authors:** Skyla R Griswold, Aaron S Brask, Krishna D Bhakta, Alexa Marie Campbell, April R Smith-Gonzalez

**Affiliations:** 1 Research, Orlando College of Osteopathic Medicine, Winter Garden, USA

**Keywords:** cranial osteopathic manipulative medicine, cranial osteopathy, osteopathic education, osteopathic physicians, pioneer in medicine

## Abstract

Dr. William Garner Sutherland was an osteopathic physician, significant for the founding of cranial osteopathy and its development into osteopathic cranial manipulative medicine techniques used today. Coming from humble beginnings, Sutherland stopped his initial education to help his struggling family and later desired to pursue an education in an effort to elevate his opportunities in society. With an education in the philosophy of osteopathy and witnessing the healing of his brother Guy Sutherland through osteopathic manipulative medicine, Dr. Sutherland sought further education under the direction of Dr. Andrew Taylor Still, M.D., D.O., founder of osteopathy. Dr. Sutherland, reflecting on his knowledge of osteopathic principles, prior foundation in mechanics and physiology, sought to understand the impact of cranial bone mobility and its associated somatic dysfunctions. Unveiling the connection between cranial bone movement and cerebrospinal fluid (CSF) flow based on primary respiratory movement (PRM) in living subjects, Dr. Sutherland contributed to osteopathic medicine development and challenged prior cranial system academic literature within the scientific community. Key objectives of this work include synthesizing the information about his formative years and educational career, examining his influences in the development of cranial osteopathy, and showcasing the effect of his legacy in modern cranial osteopathic manipulative medicine. At prestigious institutions like the Osteopathic Cranial Academy and The Sutherland Cranial Teaching Foundation, Dr. Sutherland’s approach is taught to aspiring physicians, dentists, and healthcare professionals.

## Introduction and background

Early life and education**

Dr. William Garner Sutherland was born in Portage, Wisconsin, in March of 1873. In the mid-to-late 19th century, Portage was known for simplicity and agriculture, a perfect setting for his modest family. William, the second oldest of four, was born to Robert and Dorinda Sutherland [1]. Despite working long physical hours as a blacksmith and lumberjack, his father struggled to provide for their growing family. As an empathetic and altruistic young teenager, William made the decision to halt his beloved schooling to help their struggling family. He built a relationship with a newspaper publisher, who hired William initially to be a handyman but later promoted him to chief assistant at *The Blunt Advertiser*. When the publisher left for another opportunity, William followed. This led to multiple subsequent jobs in the printing industry throughout Wisconsin, South Dakota, and Minnesota. Despite his growing résumé, William missed the academic setting and longed to continue his schooling. In the early 1890s, he was given the opportunity to enroll at Upper Iowa University in Fayette, Iowa. He enthusiastically matriculated. Details from this season of William’s life, however, are not well understood; the subjects he studied are unknown, and he dropped out shortly after classes started. Likely discouraged from this experience, William returned to the familiar printing industry and bounced between three more journalism-related jobs in the mid-1890s [2-3].

Call to the osteopathic field

As William was chasing career opportunities throughout the Midwest, he began hearing rumors of a new medical treatment called “osteopathy.” A friend of William, Herschel Connor, explained to him the osteopathic school of thought and soon thereafter enrolled in Kirksville's American School of Osteopathy. Around this same time, William’s younger brother, Guy Sutherland, received a medical diagnosis that was particularly difficult to treat with then-modern conventional approaches. Guy eventually found his way to an up-and-coming osteopathy clinic, where the treatment led to his eventual cure. The combination of Herschel’s endorsement and pursuit of osteopathy and Guy's curative osteopathic treatment rekindled William’s burning desire for higher education and gave him enough courage to once again pursue formal education, only this time in the new field of osteopathy. William followed Herschel’s footsteps and enrolled at the American School of Osteopathy in Kirksville the following year. Unbeknownst to him at the time, this was the beginning of William’s formative relationship with Dr. Andrew Taylor Still, the founder of modern-day osteopathic medicine.

Consistent with the major tenets of osteopathic philosophy today, William learned firsthand from Dr. Still about the body’s intrinsic healing capabilities when in proper alignment. It was also while studying under Dr. Still that William began theorizing and hypothesizing his own osteopathic approach to medical treatment. It is recorded that, while analyzing a particular skull specimen, William thought: “Beveled just as the gills of a fish, this hints at a flexible mobility for a respiratory mechanism.“ [4]. He continued to ponder the related anatomy and physiology throughout his training, absorbing all that he could from Dr. Still before graduating with honors in 1900.

## Review

Dr. William Garner Sutherland’s contribution to medicine 

Discovery of Cranial Mobility in the Osteopathic Field

Dr. Sutherland is widely recognized as the founder of cranial osteopathy, yet his contributions extend beyond clinical osteopathy. His interdisciplinary approach, rigorous self-experimentation, and philosophical insights contributed to the understanding of human physiology, mechanics, and holistic healing. While osteopathy served as the cornerstone of his work, Dr. Sutherland's theories, methodologies, and influence spanned diverse fields, including biomechanics, engineering, and spiritual healing.

Dr. Sutherland’s work in osteopathy was shaped by his early career in the mechanical and printing industries, where he honed an intricate understanding of structural mechanics. His capacity to observe and analyze mechanical structures led him to challenge conventional anatomical teachings, particularly the notion that cranial bones were fused and immobile in adulthood. Drawing upon principles such as leverage, tension, and joint mobility, Dr. Sutherland proposed that cranial sutures allowed for subtle micro-movements crucial for maintaining health and function.

In one of his most pioneering endeavors, Dr. Sutherland conducted self-experiments using custom-built helmets to apply pressure to his own skull, directly testing his hypothesis regarding cranial mobility. These experiments allowed him to personally experience the physiological effects of cranial bone restrictions, ultimately laying the foundation for the development of cranial osteopathy as a distinct field within osteopathic medicine.

Incorporation of Osteopathic Philosophy

Beyond anatomical and biomechanical considerations, Dr. Sutherland integrated a deeply philosophical and spiritual dimension into his work. He conceptualized an intrinsic life force, which he termed the Breath of Life, as the driving force behind the rhythmic movement of cerebrospinal fluid (CSF) and the overall vitality of the body. This concept was inspired by both scientific observation and Dr. Sutherland’s belief in the body’s innate self-regulating and healing capacity.

Dr. Sutherland’s holistic approach catalyzed the evolution of modern mind-body healing philosophies, influencing the emergence of craniosacral therapy, which extends beyond osteopathy into the realm of integrative and alternative medicine. His emphasis on gentle touch, fluid dynamics, and the subtle rhythms of the body introduced a new paradigm of health that continues to be utilized by practitioners globally.

Dr. Sutherland's impact was seen outside his immediate field, with his theories on cranial mobility and fluid dynamics challenging long-established medical beliefs. These insights prompted further research into neuroanatomy, cerebrospinal fluid circulation, and connective tissue mobility. His work influenced esteemed figures such as Dr. Harold Magoun, who helped formalize and disseminate cranial osteopathy, as well as institutions like the Sutherland Cranial Teaching Foundation, which continues to advance his methods [5-6].

Dr. William Garner Sutherland’s contributions beyond medicine 

Development of Skull Manipulative Medicine Techniques

Dr. Sutherland’s contributions looked outside the realm of osteopathic medicine, blending mechanical ingenuity, scientific exploration, and spiritual philosophy to create a transformative approach to health and healing. His pioneering exploration of cranial mobility not only established the foundational principles of cranial osteopathy but also laid the groundwork for a broader influence in the field of holistic and integrative medicine. Dr. Sutherland’s legacy serves as a testament to the power of curiosity, interdisciplinary thinking, and a deep commitment to understanding the body’s innate wisdom.

In addition to his theoretical innovations, Dr. Sutherland’s practical contributions to pediatric care set him apart from many of his contemporaries. His work in cranial manipulation as a therapeutic approach for birth trauma and developmental disorders was groundbreaking. He applied his techniques to treat children with skull deformities and neurological conditions, improving cranial mobility and promoting overall health.

Through meticulous study of skull anatomy and the pressure exerted during birth, Dr. Sutherland developed methods to release cranial restrictions that could occur during delivery, restrictions he believed could lead to long-term developmental issues. His insights into the embryology of the skull, coupled with investigations into the effects of birth pressure on cranial sutures, led to innovative treatment protocols that remain vital in modern cranial osteopathic practice. These treatments have been effective in addressing pediatric conditions such as colic, sleep disturbances, and trauma-related disorders [4].

Advocacy and Development of a New Osteopathic Model of Care

Dr. Sutherland’s advocacy for a paradigm shift in manual therapy was built upon the development of treatment techniques; he sought to redefine the approach to healthcare. He critiqued conventional medicine’s focus on symptomatic treatment, arguing that it neglected to consider the underlying causes of illness and the body’s inherent capacity for self-healing. His cranial osteopathy theory offered an alternative model that proposed cranial dysfunctions as potential root causes of a wide range of diseases and conditions-many of which had previously been difficult to treat with conventional approaches.

This therapeutic paradigm laid the foundation for a new model of care, one that emphasized prevention and the restoration of balance within the body, particularly through manual techniques aimed at enhancing fluid dynamics, such as cerebrospinal fluid circulation. Dr. Sutherland’s work has since influenced a wide array of healthcare professionals, from physical therapists to chiropractors, contributing to the global evolution of manual medicine practices [5-6].

Legacy of Dr. William Garner Sutherland

Dr. Sutherland’s Enduring Impact on Craniosacral and Osteopathic Education

Dr. Sutherland not only contributed to medical developments during his time, but he still influences how medical students and other healthcare professionals learn medicine today. He is the original creator of craniosacral work that is taught and utilized in osteopathic manipulative medicine-based professions. One of Dr. Sutherland’s greatest impacts was the formal recognition of craniosacral medicine in 1998. The Biodynamic Craniosacral Therapy Association of North America was formed to help students learn from Dr. Sutherland’s findings and development of cranial manipulative techniques for restrictive cranial somatic dysfunctions. It emphasizes biomechanical orientation, primary respiration, motion testing, and special applications. Dr. Sutherland worked with many other osteopathic professionals in order to truly explore craniosacral medicine with different perspectives and insight. 

Dr. Sutherland worked with Franklyn Sills and Dr. Randolph Stone to fully describe anatomically and medically his discoveries, ensuring sound academic literature that can be passed to aspiring healthcare practitioners. With a focus on the patient and the practitioner interaction, he describes techniques that work, associated mechanisms, and their practical clinical usage. Dr. Sutherland's theory was developed progressively over his medical career so that current therapeutic practices could be developed for high patient satisfaction and somatic dysfunction relief. His impact on the osteopathic field is taught to osteopathic students who learns cranial osteopathic practices and every doctor who treats their patients with his techniques. Dr. Sutherland’s work continues to serve as a method of practice for those seeking to learn more about the cranial system and innovate osteopathic education development [7].

Controversial Standings in the Medical Community 

Within the medical community, there has been debate as to the validity of cranial osteopathy as a practice. While cranial osteopathy techniques have been taught to healthcare professionals in osteopathic-based fields, people within the medical community have conducted studies to prove the efficacy of cranial osteopathic practices. Through growing research in cranial medicine, the contributions of Dr. Sutherland have been questioned, with people speculating whether his techniques provide genuine treatment. Some research has shown that cranial osteopathy treatment lacks a biological mechanism and diagnostic reliability. Some have even labeled it as a pseudoscience maintained between the practitioner and their patient [8]. Systematic reviews have shown that the overall effectiveness of Dr. Sutherland’s techniques is not entirely effective in conditions like infant colic, asthma, low back pain, and headache disorders. Other systematic reviews have indicated that Dr. Sutherland’s techniques are effective for conditions of chronic pain [9]. Overall, the effectiveness and anatomically based methodology of Dr. Sutherland has been and continues to be highly debated.

## Conclusions

Dr. William Garner Sutherland was a pioneer of cranial osteopathic medicine. By integrating practical applications of mechanics from early life experiences and osteopathic practices taught under the founder of osteopathy, Dr. Andrew Taylor Still, Dr. Sutherland broke prior beliefs about cranial anatomy in the scientific community, showing the relationship of cranial bone mobility and cranial physiology for promoting overall homeostatic function. Dr. Sutherland valued the principle of osteopathic philosophy in medical application and wrote literature now used in teaching curricula in healthcare institutions. His legacy continues to leave a lasting impact on medical society and osteopathic education.
